# Notes and News

**Published:** 1894-08-11

**Authors:** 


### Notes and News.

Collected and Communicated.

The Secretary of State for India has appointed Miss Annette Benson, M.D.Lond., a graduate of the London School of Medicine for Women, first physician to the Kama Hospital, Bombay, in succession to Mrs. Pechey Phipson, M.D., resigned. Miss Benson has been holding an acting appointment as resident medical officer at the Claybury Asylum, under the London County Council.

A very interesting collection of antiquities, old Roman surgical instruments and vessels used in pharmacy in ancient days, which has been brought together by Dr. Sambon on behalf of the Italian Government, was on exhibition at the Congress of the British Medical Association held last week at Bristol. To Messrs. Oppenheimer, Son, and Co. (Limited) belongs the credit of having arranged for this valuable addition to the exhibits, over which Dr. Sambon himself presided.

Last week the foundation-stone of the new Metropolitan Asylums Board Hospital at Shooter's Hill was laid by Lady Galsworthy. The new building is to accommodate 500 patients and a staff of some 270 persons. It is the first of the three great hospitals for infectious diseases which are to be erected on the south side of London by the Asylums Board, and the estimated cost, including drains, roads, yards, and boundary walls, is about £200,000.

The Church Sanitary Association held its second annual meeting on Thursday, July 26th, by the permission of the Duke of Westminster, at Grosvenor House, Sir Joseph Fayrer presiding. The aim of this association is to enlist all earnest Church workers in a common effort to teach the laws of health from the pulpit and in the schools, an aim which will have the hearty co-operation of all sensible people. During the past year application has been made to the Home Secretary, asking for the creation of a Ministry of Health, and twenty-four meetings have been held in London and in the country. Sir Spencer Wells, Bart., Sir Benjamin Ward Richardson, Bishop Mitchinson, and Dr. Norman Kerr took part in the proceedings.

Between the dates July 29th and August 4th, 313 cases of cholera are said to have occurred in St. Petersburg, the deaths numbering 240.

The new Convalescent Home in Charnwood Forest, Leicester, was formally opened by the Duke of Rutland on Wednesday last.

The first Congress of Indian Medical Men is to be held at Calcutta this winter—from December 24th to 29th. No doubt many doctors of all nationalities will be glad to avail themselves of the invitation of the executive committee to be present.

The Committee of the Central London Throat and Ear Hospital, Gray's Inn Road, have adopted the plans of Mr. Ernest Turner, F.R.I.B.A., for a new out-patient department and nurses' quarters, and have entered into a contract with Messrs. J. Simpson and Sons for the erection of the buildings.

The Lord Mayor is appealing for funds to enable the Mansion House Council on the Dwellings of the Poor to carry on its good work. Subscriptions may be sent either to the Lord Mayor, at the Mansion House, or to the Hon. Secretary, at 31, Imperial Buildings, Ludgate Circus.

An American contemporary states that a medical woman, Dr. Lilias Hamilton, has lately gone to Kabul in the capacity of physician to the Harem of the Ameer of Afghanistan, Abdurrahman. A guard of six soldiers is told off for her protection, of whom three are always to accompany her when she goes about the city. The Indian Government considers the undertaking a risky one, and has endeavoured to dissuade Dr. Hamilton from her venture, at the same time disclaiming all responsibility should any harm befall her.

The Birmingham Hospital Saturday Fund has closed its banking account for this year, and the total receipts are as follows: Manufactories, £11,707 5s. 7d.; street collections, £426 8s. 10d.; Grand Theatre and Oberon Minstrels, £128; total, £12,261 14s. 5d. Mr. J. C. Holder has generously made up this amount to £12,500 as against the sum of £11,749 13s., the result of last year's collection. A revised scheme for the distribution of the fund was laid before a special meeting of the board and delegates the other day by Mr. Smedley, and was adopted with unanimous approval. Twenty-one years ago 64 per cent. of the entire proceeds was given to the General and Queen's Hospitals, last year only 42 per cent. was allowed them. They received, in fact, 50 per cent. less last year, in proportion to the work done, than in 1872. Under the new plan the General Hospital will now receive £3,000, instead of £2,907; the Queen's Hospital £2,000, as against £1,600; the Children's Hospital £750, instead of £709; whilst in the case of the Eye Hospital, which has a surplus every year, £600 will be awarded, instead of £793. These are only a few instances. The awards have been revised throughout, and it will be seen that a far more equable plan of distribution has been arrived at as the result.

